# Comparative Analysis of rAAV Production from Plasmid-Encoded Versus Chromosomally Integrated rAAV Transgene in HEK293 Cells

**DOI:** 10.3390/ijms27125538

**Published:** 2026-06-18

**Authors:** Maria Toth, Anastasia Rempe, Georg Smesnik, Manuel Reithofer, Astrid Dürauer, Reingard Grabherr

**Affiliations:** 1 Christian Doppler Laboratory on Knowledge-Based Production of Gene Therapy Vectors, Institute of Bioprocess Science and Engineering, Department of Biotechnology and Food Science, BOKU University, 1190 Vienna, Austria; maria.toth@boku.ac.at (M.T.); anastasia.rempe@boku.ac.at (A.R.); georg.smesnik@boku.ac.at (G.S.); astrid.duerauer@boku.ac.at (A.D.); 2 Institute of Molecular Biotechnology, Department of Biotechnology and Food Science, BOKU University, 1190 Vienna, Austria; manuel.reithofer@boku.ac.at

**Keywords:** rAAV, Adenovirus, gene therapy, HEK293, stable cell lines, RMCE, GOI, backbone packaging, product-related-impurities, potency

## Abstract

Stable cell lines have recently achieved recombinant adeno-associated virus (rAAV) titers comparable to the standard triple transfection approach, making them a promising alternative to plasmid-based production systems. However, whether integration of the rAAV transgene into the host genome influences packaging efficiency and vector quality remains unclear. In this study, we generated stable HEK293 cell lines carrying the rAAV transgene in their genome. rAAV production was enabled by supplying the rep/cap and helper genes on two plasmids, rendering vector genome generation dependent on the chromosomally integrated transgene. Although the stable cell lines produced a 4.5-fold lower titer of viral genomes (VGs) compared to the standard triple transfection method, VG-normalized potency was four times higher. Detailed particle characterization further revealed 3-fold lower plasmid backbone DNA packaging in rAAVs produced by stable cell lines relative to triple transfection. Consistent results were obtained from mass photometry and ELISA/ddPCR analyses for the double transfection condition, while discrepancies emerged under triple transfection. These findings emphasize the importance of functional and qualitative assessments for evaluating different rAAV production approaches.

## 1. Introduction

Recombinant adeno-associated virus (rAAV) vectors hold substantial promise for in vivo gene therapy, as they support long-term transgene expression [1,2,3]. rAAVs transduce non-dividing cells where their DNA persists predominantly in the form of episomes, posing an improved safety profile in terms of insertional mutagenesis compared to integrating vectors [4]. In addition, there is increasing evidence that rAAVs integrate into the genome at low frequencies, which can drive transgene expression [5,6,7]. Various tissues like the eye, liver, central nervous system, or muscles can be targeted depending on the rAAV serotype administered [1,2,3,8,9]. Several approved clinical rAAV products for diseases like hemophilia or spinal muscular atrophy are on the market, highlighting its translational relevance [10,11,12]. Despite these strengths, the manufacturing of rAAVs as gene therapy vectors is costly and challenging at clinical scale. Production is often inefficient, with expensive raw materials, complex upstream processes, batch-to-batch variability and high therapeutic doses [13,14,15,16]. Generated particles entail a large proportion of empty, partially, and overfilled rAAVs [17,18]. Furthermore, undesired entities such as rAAVs containing viral genome fragments or off-target DNA (including bacterial DNA, host cell DNA, and chimera of host cell DNA and rAAV transgene) cannot be efficiently depleted by state-of-the-art chromatographic purification and thus require monitoring of each batch by extensive analytics [17,18,19,20,21].

rAAVs are conventionally produced by transiently transfecting HEK293 cells with three plasmids: one encoding the artificial rAAV genome (rAAV transgene), a second carrying the AAV genes *rep* and *cap*, and a third providing adenoviral helper functions (E2A, E4, 22K, 33K, and VA RNA) [22,23,24]. During the establishment of the HEK293 cell line, the adenoviral E1 gene was stably integrated into the HEK293 genome [25,26]. Triple transfection is currently the state-of-the-art production method. Alternatively, a more modern approach is a double transfection, where rAAV transgene and RepCap are combined into one plasmid [27,28]. Transfection processes rely on large-scale plasmid production and can result in unintended packaging of plasmid DNA into the final particles [16,19,29].

During rAAV production, fragments of the bacterial plasmid backbone are frequently packaged into rAAV capsids, sometimes constituting up to 10% of produced filled particles [21,30,31,32]. These backbone sequences often include antibiotic resistance genes, bacterial replication origins, and other non-mammalian regulatory elements. Bacterial DNA contains unmethylated CpGs, which are recognized as pathogen-associated molecular patterns that activate immune response and are deleterious to long-term gene expression [33,34,35,36]. This was shown in a study done by Keiser et al. on non-human primates (NHPs), where the 3′-inverted terminal repeat (ITR) showcased substantial promoter activity, resulting in the transcription of open reading frames in plasmid backbone DNA [33]. Three months after administration, NHP exhibited signs of inflammatory response and reduced transgene expression. As reported by Keiser et al., this toxicity was not observed in previous mice studies. Furthermore, rAAVs have been shown to integrate into the genome of hepatocytes, including the bacterial ampicillin resistance gene, resulting in possibly genotoxic or oncogenic events [5,6,7,29,33]. To mitigate these risks, vector designs can be optimized by maximizing the distance between ITRs and bacterial elements with insulators or “safe” human-like CpG free backbone sequences. Another strategy is to completely eliminate backbone sequences by using synthetic DNA such as closed-end linear or minicircle DNA [21,35,37].

As an alternative to plasmid transfection in HEK293 cells, stable cell lines can be developed which carry the necessary AAV and adenoviral genes, as well as the rAAV transgene. These viral genes are toxic and thus require tight gene regulation by inducible expression systems [38,39,40,41,42]. Work by Lin et al. showed that initial stable cell lines carrying all necessary genes exhibited low rAAV titers. Productivity was elevated to the level of the triple transfection benchmark by increasing the integration copy number of *cap* and small *rep* genes, thereby identifying them as limiting factors [43]. Stable producer cell lines are competitive with triple transfection in terms of yield, while eliminating the need for expensive plasmid DNA and the variability of transfection [16,43].

The question remains whether the packaging efficiency and accuracy is affected by stable integration of the rAAV transgene. So far, rAAV production in stable cell lines is commonly done by the integration of all necessary genes at once [42]. In the present work, the impact of rAAV transgene integration on rAAV particle quality was assessed. Therefore, stable HEK293 cell lines were generated, carrying the rAAV transgene in their genome. rAAV production was evaluated by transiently supplying *rep*, *cap*, and adenoviral helper functions via plasmid transfection. This was compared to the state-of-the-art triple transfection approach, in which all components, including the rAAV transgene, were provided via plasmids. Viral genome production in stable cell lines was lower than in classical triple transfection. However, produced particles from stable clones contained less plasmid backbone DNA and exhibited a 4-fold increase in viral genome (VG)-normalized potency, suggesting that genomic integration of the rAAV transgene can improve vector quality.

## 2. Results

### 2.1. Characterization of Stable HEK293 Cell Lines

**Figure 1:**
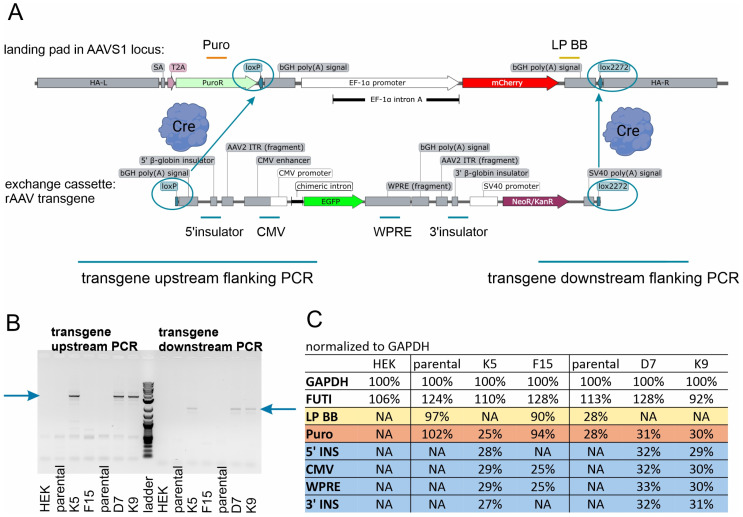
Characterization of stable cell lines carrying an rAAV transgene. (**A**) Landing pad in AAVS1 locus carrying mCherry and exchange cassette with rAAV transgene. rAAV transgene cassette was integrated using the Cre-loxP RMCE, visualized by the arrows. rAAV transgene reaches from ITR to ITR and is flanked by genomic insulators. The rAAV transgene carries eGFP under a CMV promoter and enhancer together with the WPRE translational enhancer. Primer pairs used for PCR and ddPCR depicted by colored lines. (**B**) Flanking PCR to assess site-specific integration in genetically unmodified HEK293 cells, parental HEK293 cells harboring the loxP landing pad, and four derived stable clones carrying the exchange cassette. One primer annealed in the unmodified genomic region and the other within the introduced cassette. Expected amplicons were 3650 bp (transgene upstream) and 2000 bp (transgene downstream) and are indicated by arrows. (**C**) Digital droplet PCR (ddPCR) on genomic DNA in technical triplicates (*n* = 3) to assess integration copy number. Two housekeeping genes were included and values were normalized to GAPDH and expressed as percent. Primer set, indicated by colored lines in (**A**), targeted the landing pad backbone (LP BB; yellow), puromycin resistance (Puro; orange) that amplifies in both the landing pad and the integrated cassette, and four regions specific to the integration cassette (blue).

Stable cell lines carrying the rAAV transgene were generated using recombinase-mediated cassette exchange (RMCE). Therefore, HEK293 cell lines were constructed carrying a landing pad by transfecting a Cas9 plasmid with a guide RNA for the AAVS1 locus. The landing pad contains a splice acceptor (SA), followed by a T2A self-cleaving sequence, a puromycin resistance gene, loxP sites, and mCherry as a reporter (Figure 1A). This landing pad was integrated into the AAVS1 locus, the natural integration site for wild-type AAV [44]. After antibiotic selection, single cell sorting and characterization, the cassette exchange was performed. Co-transfection of a Cre recombinase expressing plasmid and the exchange cassette enabled site-specific integration of the rAAV transgene at AAVS1, resulting in an engineered cell line containing the expression cassette. The rAAV transgene spanned from ITR to ITR and was flanked by β-globin insulators. The transgene eGFP was controlled by a CMV promoter and enhancer as well as a Woodchuck Hepatitis Virus Posttranscriptional Regulatory Element (WPRE) translational enhancer, resulting in high protein expression. Additionally, a neomycin resistance gene controlled by a SV40 promoter was located at the 3′ end of the integration cassette. Single cell sorting was performed for mCherry negative and eGFP positive cells (Figure S1A).

Cassette exchange was performed with two separate parental clones. K5 and F15 originated from one parent, and D7 and K9 from the other. The correct location and orientation of the integrated cassette was assessed by PCR screenings on each end of the integration site. As shown in Figure 1B, unmodified HEK293 cells and the parental cell lines yielded no PCR product. Screening of K5, D7, and K9 resulted in a band of the expected size, whereas F15 did not.

To determine the copy number of integrated fragments, genomic DNA (gDNA) was isolated and analyzed by ddPCR with various primer pairs targeting different regions of the landing pad or integration cassette (Figure 1C). The obtained concentrations were normalized to the housekeeping gene glyceraldehyde 3-phosphate dehydrogenase (GAPDH) and reported as percent. Non-modified HEK293 cells served as a negative control and ddPCR was below the detection limit with primer pairs specific to the landing pad or integration cassette. Both parental cell lines showed presence of the landing pad with the primer pairs targeting the landing pad backbone (LP BB) and the puromycin resistance gene (Puro), while cassette-specific primer pairs (Figure 1C in blue) yielded no PCR product. The parental cell line of K5 and F15 showed a higher amount of landing pad (around 100%) than the parental cell line for D7 and K9 (around 30%). F15 presented similar amounts of LP BB and Puro as the parental cell line, indicating the presence of the landing pad. Elements of the integration cassette were detectable as tested for the CMV promoter and the WPRE, whereas genomic insulators were absent. This was consistent with a partial random integration rather than site-specific RMCE. In contrast, K5, D7, and K9 showed uniform genomic profiles with each primer pair at approximately 30%. Assuming a triploid karyotype for HEK293, this corresponds to an integration copy number of 1 [26]. Flanking PCR and ddPCR results for these three clones support a site-specific genome integration event. Therefore, the three clones were used for further experiments. Additionally, gDNA was analyzed again, after all experiments were finished (after 40 passages), proving that the genomic profile was stable (Figure S1B).

### 2.2. Plasmid-Based rAAV Production

**Figure 2:**
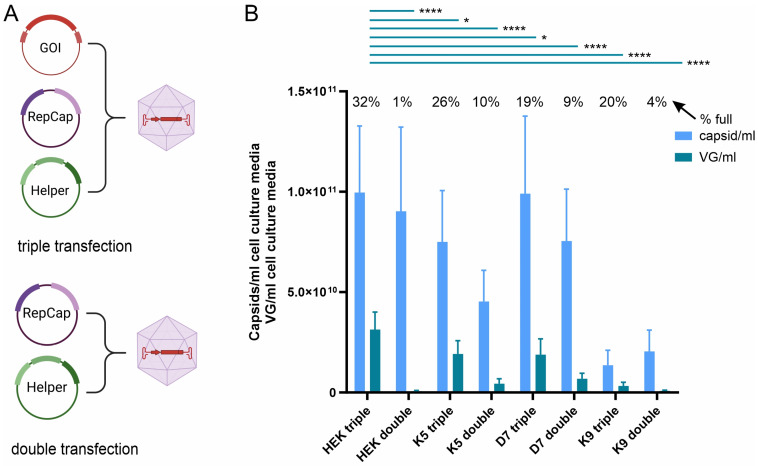
Plasmid-based rAAV8 production with HEK293 cells and three stable cell lines carrying the rAAV transgene. (**A**) Depicts the experimental design. All cells were transfected with the three plasmids GOI, Rep2Cap8, and helper, which is the standard setting for rAAV production, referred to as triple transfection. The GOI plasmid was not provided in the double transfection as the rAAV transgene was integrated into the genome of the stable cells. (**B**) Production outcomes for HEK293 cell line and three genome-integrated clones under both conditions. After 72 h of production, capsids were measured with ELISA (Progen) and viral genome (VG) titers were obtained by ddPCR and expressed per ml cell culture media. % full represents the percentage of rAAV particles filled with a viral genome calculated with the means of capsid and VG titers. The column and error bars represent the mean and SD of *n* = 4 biological replicates for K9 and *n* = 5 for the remaining samples. Data was evaluated using one-way ANOVA with Tukey’s post-test. The statistical significance of VG titers are given by * and ****, which represent *p* ≤ 0.05 and ≤0.0001 respectively.

rAAV production was evaluated under two conditions. For triple transfection, a gene of interest (GOI) plasmid carrying the same rAAV transgene as integrated into the stable clones was co-transfected with a Rep2Cap8 plasmid for rAAV8 production and a helper plasmid, providing the necessary adenoviral helper factors (Figure 2A). Particle production was compared between triple and double transfection, where Rep2Cap8 and helper were co-transfected. In the double transfection setup, the viral genome to be packaged was only present when amplified from the chromosomal integration site. An original HEK293 cell line was used as a control and compared to the three stably engineered clones K5, D7, and K9 with the integrated rAAV transgene.

For the unmodified HEK293 cell line, the triple transfection resulted in the highest titers with a titer of 1.0 × 10^11^ capsids/mL, 3.1 × 10^10^ VG/mL, and 32% filled particles. Double transfection yielded predominantly empty particles, reflected in 9.0 × 10^10^ capsids/mL and 1% filled capsids. K5 and D7 showed high titers for triple transfection (7.5 and 9.9 × 10^10^ capsids/mL for K5 and D7), albeit viral genome production (1.9 and 1.9 × 10^10^ VG/mL for K5 and D7) was statistically lower than triple transfection in the HEK293 cell line (*p* = 0.0158 for K5 and *p* = 0.0117 for D7). This was reflected in the lower amounts of filled particles as well (26% and 19% respectively). Clone K9 displayed 7-fold lower capsid titers than the D7 clone.

After double transfection, K5 and D7 reached capsid titers not significantly different from HEK293 triple transfection (4.5 and 7.5 × 10^10^ capsids/mL for K5 and D7), but with reduced viral genomes titers relative to triple transfection (4.4 and 6.8 × 10^9^ VG/mL for K5 and D7), which was consistent with lower filled fractions (9–10%). K9 produced 4% filled rAAVs and lower titers compared to K5 and D7. Overall, viral genome production seemed to be reduced for the stable clones compared to unmodified HEK293 cells. As eGFP expression from the rAAV transgene was very strong, we hypothesized that this over-expression hampered rAAV production. Therefore, downregulation of eGFP translation by the addition of specific siRNA and its impact on rAAV production was explored. The co-transfected siRNA reduced eGFP expression, as visualized in Figure S3A. However, the siRNA was transfected with RNAiMAX, a derivative of lipofectamine, which severely reduced rAAV production and caused a lot of variability in the biological replicates (Figure S3B).

### 2.3. rAAV Production with Helper Virus

**Figure 3:**
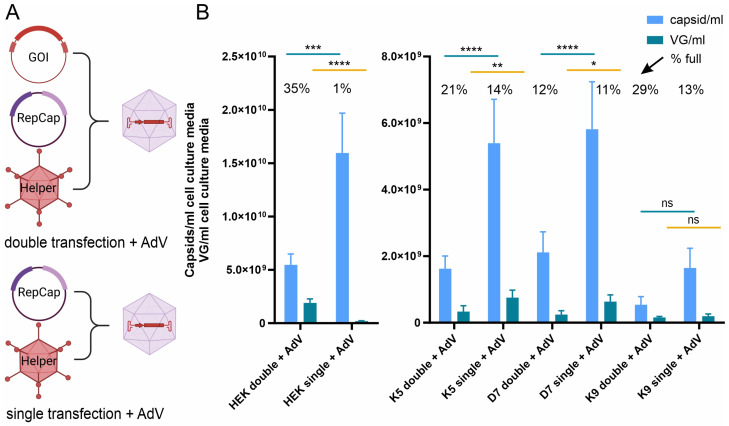
Adenovirus-assisted rAAV8 production in HEK293 cells and three stable cell lines carrying the rAAV transgene. (**A**) Illustration of experimental design. In the double transfection + AdV condition, the GOI plasmid was co-transfected with Rep2Cap8. An Adenovirus AdV-ΔE3-MLP-eGFP with a deleted E3 gene and eGFP under the major late promoter provided the helper factors. The GOI plasmid was omitted for the single transfection, where Rep2Cap8 only was transfected and AdV-ΔE3-MLP-eGFP was used as a helper virus. Here, viral genome production was dependent on the chromosomal presence of the rAAV transgene in stable cell lines. (**B**) Production titers across HEK293 cells and the three stable clones. Panels use different y-axis scales for easier visualization. Capsids were measured by ELISA and viral genomes (VGs) by ddPCR and expressed per ml cell culture media. The full to empty ratio was calculated with the means of capsid and viral genome titers and indicated in percentage above the bars. The column and error bars represent the mean and SD of *n* = 4 biological replicates for K9 and *n* = 5 for the remaining samples. Data was evaluated using one-way ANOVA with Tukey’s post-test. The statistical significance of capsids (light blue) and VG (orange) titers are given by * (*p* ≤ 0.05), ** (*p* ≤ 0.01), *** (*p* ≤ 0.001), and **** (*p* ≤ 0.0001) respectively. ns—not significant.

AdV was used instead of the helper plasmid to explore viral genome production from chromosomal integration and plasmid. The AdV-ΔE3-MLP-eGFP has the E3 gene deleted and eGFP under control of the major late promoter (MLP). Thus, more adenoviral factors were provided to the cells during rAAV production. Here, a similar experimental setup was used as before, visualized in Figure 3A. The GOI plasmid, carrying the same rAAV transgene as integrated into the stable cell lines, was co-transfected with the Rep2Cap8 plasmid and the AdV provided the necessary helper factors for rAAV production (double transfection + AdV). This was compared to a single transfection of the Rep2Cap8 plasmid only in combination with the AdV infection.

Overall obtained titers were lower than the plasmid-based rAAV productions (Figure 3B). The HEK293 cell line yielded 5.5 × 10^9^ capsids/mL for double transfection + AdV, whereas 2.9-fold more capsids were produced in the single transfection + AdV (1.6 × 10^10^ capsids/mL). Furthermore, similar percentages of filled particles were obtained for plasmid-based and helper virus mediated rAAV production in the HEK293 cell line (see Figure 2B). The stable cell lines K5, D7, and K9 produced 21, 12, and 29% filled particles respectively in double transfection + AdV and were shown in a separate graph for better visualization. For HEK293 cells, K5 and D7 capsid titers were significantly higher (*p* ≤ 0.001) in the single as compared to the double transfection + AdV, resulting in 3-fold differences in capsid titers. Double and single transfection + AdV yielded 1.6 and 5.4 × 10^9^ capsids/mL for K5 and 2.1 and 5.8 × 10^9^ capsids/mL for D7. K5 (*p* = 0.0050) and D7 (*p* = 0.0100) showed a significant increase in viral genome titer for the single transfection + AdV compared to double transfection + AdV (3.4 and 7.5 × 10^8^ viral genomes/mL for K5 and 2.5 and 6.3 × 10^8^ viral genomes/mL for D7). The fraction of filled particles in single compared to double transfection + AdV decreased to 14% for K5, whereas D7 showed a reduction by 1 point to 11%. K9 showed no significant differences between conditions. As the AdV has eGFP under the control of the MLP, eGFP expression levels were rather high. Therefore, as for plasmid-based rAAV production, an siRNA was transfected to explore the reduction of eGFP expression. As observed with the plasmid-based rAAV production, variability increased with the transfection of the siRNA (Figure S3C,D).

### 2.4. In-Depth Characterization of Produced rAAVs

**Figure 4:**
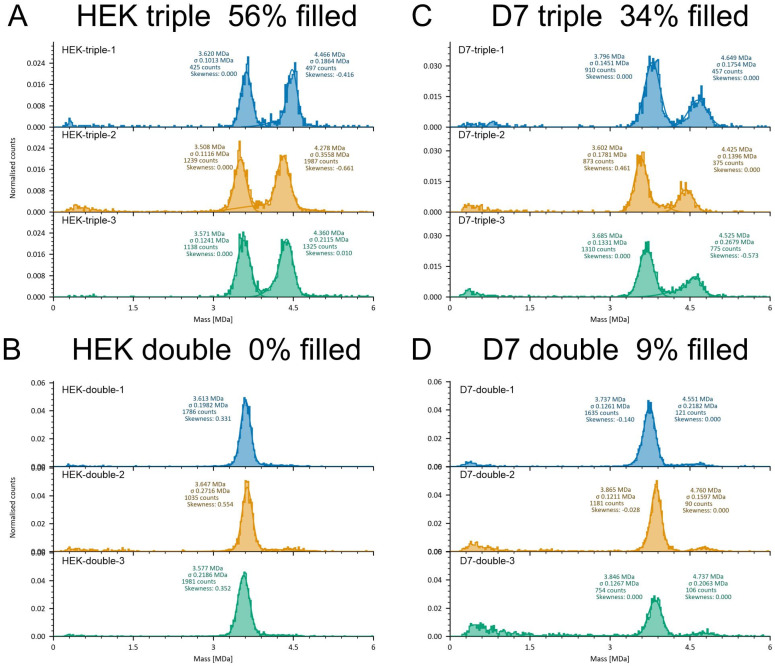
Mass photometry of purified rAAVs obtained from plasmid-based rAAV production with SamuxMP (Refeyn). *n* = 3 biological replicates were measured for each condition and peaks were evaluated using Gaussian peak fitting, resulting in percent filled rAAVs (mean listed next to heading). (**A**) HEK293 triple and (**B**) double transfection were compared to the stable cell line D7 carrying the rAAV transgene (**C**) triple and (**D**) double transfection.

rAAVs from plasmid-based rAAV production (see Figure 2) were purified using an AAVX resin and measured by mass photometry in biological triplicates. This allowed for determination of an orthogonal full to empty ratio and the assessment of possibly overfilled rAAV particles. rAAVs from triple transfection in HEK293 cells resulted in mean ± standard deviation 56% ± 4% filled particles whereas the HEK293 double transfection showed no detectable peak for filled particles (Figure 4). Triple transfection with the D7 clone yielded 34% ± 4%, and 9% ± 3% was obtained from the double transfection. Comparing this to the ELISA and ddPCR data shown in Figure 2B, the full to empty ratios aligned for both HEK293 and D7 double transfections. With triple transfections, the mass photometer showed higher percentages of filled particles as compared to the ratio resulting from ELISA and ddPCR analyses.

Given that ddPCR is a sequence-specific method and the mass photometer is a physical method based on the detection of mass, the question remains whether DNA other than the desired rAAV transgene was packaged into rAAVs. The discrepancy between the mass photometry and ddPCR was most prominent for the triple transfection condition, where the GOI was provided on a plasmid as opposed to the double transfection setting. Therefore, a ddPCR method was setup to investigate the presence of backbone DNA in rAAVs. As the samples have been purified with AAVX resin prior to DNA isolation, the possibility of contaminating plasmid DNA from transfection was low.

**Figure 5:**
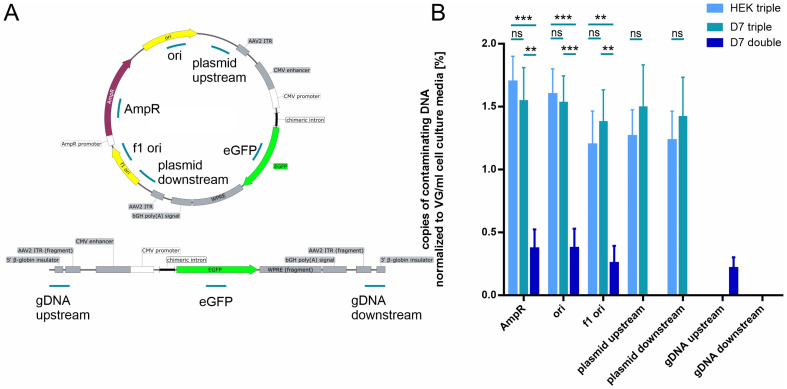
ddPCR to detect unspecific DNA packaging in purified rAAV particles. (**A**) Schematic representation of primer pairs for ampicillin resistance (AmpR), ori, and f1 ori to detect plasmid backbone DNA. These sequences are present in Rep2Cap8 and helper plasmids too. Additionally, the upstream and downstream regions adjacent to rAAV ITRs on the plasmid were analyzed (plasmid upstream and downstream). Similarly, the upstream and downstream regions for the genome-integrated rAAV transgene, which are genomic insulators (gDNA upstream and downstream) and unique to the integration cassette, were evaluated. (**B**) Contaminating backbone DNA in rAAVs produced by HEK293 triple transfection and D7 triple and double transfection. The obtained copies/µL of the ddPCR were normalized to the respective eGFP titer representing the amount of contaminating DNA per viral genome, expressed as percent. *n* = 3 biological replicates were measured in *n* = 3 technical replicates by ddPCR. Mean and SD are depicted as columns and error bars. Data was evaluated using Student’s *t*-test. The statistical significant difference between HEK triple transfection and D7 double transfection are given by ** (≤0.01) and *** (≤0.001) respectively. ns—not significant.

rAAVs produced from original HEK293 cells after triple transfection showed presence of plasmid backbone DNA from 1.2% up to 1.7% in the particles (see Figure 5). Regions directly adjacent to the ITRs were found in similar concentrations, indicating the packaging of the entire plasmid backbone. As expected, sequences specific for the chromosomally integrated transgene were absent in these particles. rAAVs from triple transfection of the D7 clone showed a similar plasmid contamination profile as HEK293 triple transfection. gDNA of the transgene upstream region was not found in rAAV genomes. Double transfection of D7 revealed a 3-fold reduction in packaged plasmid backbone DNA compared to triple transfection in HEK293, even though these sequences were present in Rep2Cap8 as well as helper plasmids. Plasmid backbone DNA was found up to 0.4% and sequences directly adjacent to the ITRs in the genome were found at 0.2% for transgene upstream end. gDNA of the transgene downstream sequence was not detected.

**Figure 6:**
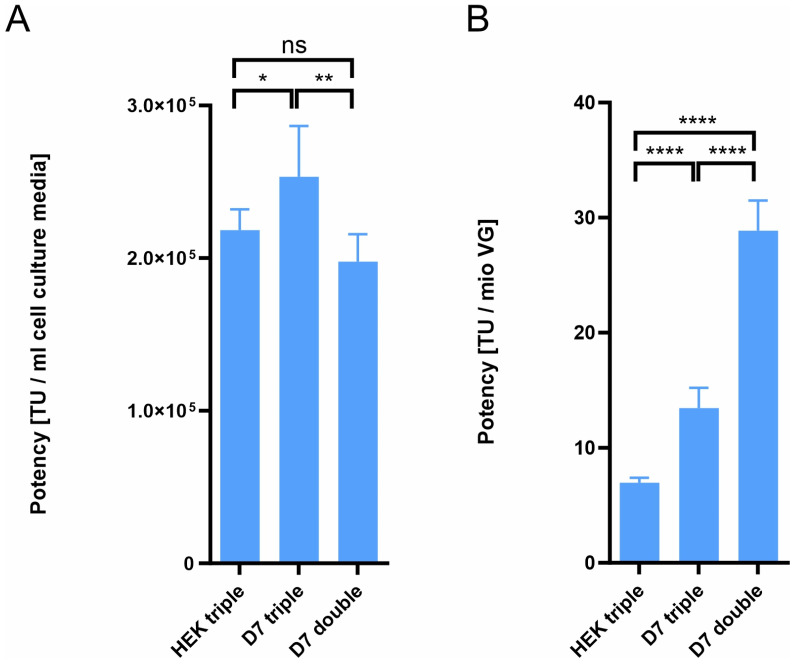
Potency assay with purified rAAVs from HEK293 and D7 triple transfection and D7 double transfection. Different concentrations of rAAVs carrying a CMV-eGFP-WPRE sequence were incubated with HEK293 cells for 48 h. Afterwards, cells were measured with flow cytometry. VG/cell dosages corresponding to 5–25% fluorescent cells were taken for potency evaluation, corresponding to the linear range of the assay. (**A**) Based on percent fluorescent cells, transducing units/mL cell culture media (TU/mL) were calculated. (**B**) As the sample had different rAAV concentrations, the transducing titers were normalized to the viral genome titers and expressed transducing units per million viral genomes (TU/mio VG). The column and error bars represent the mean and SD of *n* = 6 replicates. Data was evaluated using one-way ANOVA with Tukey’s post-test. The statistical significance is given by * (*p* ≤ 0.05), ** (*p* ≤ 0.01), and **** (*p* ≤ 0.0001). ns—not significant.

Bioactivity was assessed by a potency assay where a defined number of viral genomes per cell was incubated with HEK293 cells for 48 h. As the rAAVs carry a CMV-eGFP-WPRE expression cassette, apparently transduced cells were detectable by eGFP fluorescence via flow cytometry. Percentage of eGFP positive cells and geometric mean fluorescence intensity as a function of VG/cell are depicted in Figure S4. VG/cell dosages, resulting in 5–25% eGFP positive cells, were used for potency evaluation. This corresponds to the linear range of the assay. The evaluated VG dosages were 1.0 and 2.5 × 10^4^ VG/cell for HEK293 triple transfection, 4.0 × 10^3^ and 1.0 × 10^4^ VG/cell for D7 triple transfection and 2.0 and 4.0 × 10^3^ VG/cell for D7 double transfection. Potency given as transducing units per ml cell culture media (TU/mL) is shown in Figure 6A. rAAVs from chromosomally provided rAAV transgene in D7 and triple transfection in HEK293 produce similar TU/mL in production while volumetric VG titer was reduced 4.5-fold for D7 double transfection (Figure 2B). rAAVs from the D7 clone with triple transfection showed elevated volumetric potency over HEK293 triple transfection.

Furthermore, potency was normalized to the VG concentration of the sample used, resulting in potency as transducing units/million viral genomes (TU/mio VG). This unit reflects transducing particles per one million viral genomes. rAAVs produced in HEK293 cells by triple transfection showed the lowest potency of mean ± SD 7.0 ± 0.4 TU/mio VG (see Figure 6). An increased potency with 13.4 ± 1.8 TU/mio VG was detected for rAAVs harvested from clone D7 after triple transfection. D7 double transfection resulted in the highest potency of 28.9 ± 2.6 TU/mio VG and poses a 4-fold increase compared to HEK293 triple transfection. These results showed significant differences (*p* ≤ 0.001) in the bioactivity of produced rAAVs and highlight that obtained capsids and VG titers do not necessarily reflect the functional output of these bionanoparticles.

## 3. Discussion

Triple plasmid transfection of suitable mammalian cell lines is a widely used method for producing rAAVs; however, it presents several challenges throughout the production process. A key limitation is that only cells successfully transfected with all three plasmids can generate complete and fully packaged rAAV particles [14]. To overcome this, various strategies have been explored, including the stable integration of some or all required genetic elements into the host cell genome to create packaging cell lines. Several factors must be considered in such systems. For example, proteins such as Rep78 are cytotoxic and can inhibit cell growth. Moreover, genomic integration generally results in lower copy numbers compared to transient plasmid transfection, which can negatively affect overall yield [39,43]. Ultimately, the quality of the final rAAV product depends on several factors, including efficient genome packaging, a high full to empty capsid ratio, and minimal contamination with non-target DNA within the recombinant rAAV particles [17,19,21,33,35].

wtAAV generally packages its own genome more efficiently than rAAV, most likely because the wild-type genome is the natural size and structure the virus evolved to package [45]. rAAV often shows lower packaging efficiency, especially when the vector genome is close to the upper size limit, and empty or defective capsids are more common [46,47]. This study aimed to assess the impact of an integrated rAAV transgene in the context of rAAV production. Therefore, the effect of stably integrating the rAAV transgene into the HEK293 genome as opposed to plasmid-derived rAAV transgene was evaluated in terms of rAAV packaging efficiency and product quality rather than quantitative titers.

The rAAV transgene, represented by the reporter gene eGFP, was integrated into HEK293 cells using the Cre-loxP-based RMCE method after having inserted a suitable landing pad into the safe harbor AAVS1. Despite flow cytometry-assisted single cell sorting, one of the four characterized clones displayed a random partial genome integration rather than the intended site-specific insertion. However, for the remaining clones K5, D7, and K9, flanking PCR and ddPCR data supports a site-specific genome integration event. The two parental clones used for RMCE differed in the copy number of the landing pad by a factor of 3, yet all obtained clones displayed a presence of the integration cassette of around 30% relative to GAPDH as measured by ddPCR (Figure 1C). Assuming a triploid state of the HEK293 cell line, this indicates a copy number of one [26].

During plasmid-based rAAV production the provision of the rAAV transgene via plasmid versus chromosomal integration was compared. This revealed that viral genome production was dependent on the initial copy number of viral genomes present at the time of transfection. Plasmid transfection delivers vast amounts of copies of the GOI plasmid to the cell. Compared to this the rAAV transgene was present with a copy number of one in the chromosomes of our tested stable cell lines. As shown in Figure 2B, the viral genome titer and percent filled rAAVs of D7 with the double transfection was approximately 4-fold lower than measured after HEK293 triple transfection. Work by Zhang and colleagues showed that direct packaging of plasmid DNA into rAAVs can happen, as opposed to synthesis of viral genomes by the cell [48]. J. Fraser Wright even proposed that the amount of transfected GOI plasmids is comparable to the produced viral genomes and direct packaging of plasmid DNA should be considered as a way that rAAVs are being filled with DNA [49].

Given the lower transgene copy number in stable clones, efficient viral genome amplification is essential. New adenoviral helper factors aiding rAAV production are still being identified and AdV is a useful tool in basic research to assess the production of rAAVs [50]. As an exploratory experiment, rAAV production with an E3-deleted AdV was investigated to mimic a natural AdV-assisted rescue of the rAAV transgene from the genome. The E3 gene of the AdV is involved in immunomodulation but is not necessary for AdV production in cell culture. However, strong innate immune responses to AdV have been observed without the E3 gene [51,52]. In our experiments, rAAV titers were strongly reduced when the cells were infected with AdV compared to the plasmid-based production. This could be due to the missing E3 gene in the AdV and a strong immune response in the cells or due to the delicate timing of the expression of helper factors. Because the transfection agent PEIpro hindered AdV entry, infection was performed two hours before transfection, potentially altering helper factor kinetics relative to the plasmid-only setup. Nevertheless, rAAV titers in single transfection + AdV exceeded those in double transfection + AdV for the stable lines (Figure 3B). This observation indicates that adenoviral helper factors may impact viral genome amplification. However, further studies are necessary to understand the observed effects.

Out of the three stable clones assessed in rAAV production, D7 produced the highest viral genome titers and was therefore chosen for further in-depth rAAV characterization. Therefore, HEK293 cells and the D7 cell line were used to generate purified rAAVs with triple and double transfection, respectively. Mass photometry revealed that triple transfections showed higher full to empty ratios as compared to ddPCR and ELISA results, whereas for double transfection, the results aligned well. As the difference between triple and double transfection is the provision of the GOI on a plasmid versus chromosomally, we suspected the packaging of backbone DNA, which would be measured by mass photometry but not by GOI-specific ddPCR. A targeted ddPCR confirmed over 1% of plasmid backbone packaging in rAAVs after HEK293 and D7 triple transfection, whereas D7 double transfection showed ~4-fold lower presence of contaminating plasmid DNA in the particles. Additionally, 1% of backbone DNA is in accordance with reported contaminations in the literature [21,30,31,32]. Plasmid DNA is preferentially packaged when it is close to Rep binding sites, which are located in ITRs and the p5 promoter [35]. However, 1% plasmid backbone packaging does not account for the discrepancy observed between mass photometry and ddPCR. Host cell DNA can also be packaged into rAAVs, which could be analyzed with rAAV sequencing, which in this study, was not feasible due to limited titers from D7 double transfection. Mass photometry is a physical detection method based on differences in mass, whereas ddPCR is a sequence-specific method. Therefore, a difference in the obtained percentage of full particles is to be expected, which should be accounted for by rAAVs carrying backbone or host cell DNA. The choice of detection method for upstream samples purified by batch chromatography should be made with caution, as differences in analytical principles can affect estimates of full to empty ratios and orthogonal assays should be employed to ensure robust interpretation.

Volumetric potency (Figure 6A) in terms of TU/mL was similar between rAAV production from chromosomal transgene of the stable clone D7 and plasmid-based production in original HEK293 cells. However, D7 double transfection has a 4.5-fold lower VG titer and therefore a 4.1-fold higher VG-normalized potency compared to HEK293 triple transfection. This highlights the importance of assessing functional titers as opposed to physical titers when evaluating rAAV production with profound differences in the production setup.

This study aimed at assessing the impact of integrating the rAAV transgene rather than to improve rAAV production in terms of yield. The integration of an rAAV transgene carrying CMV-eGFP revealed increased VG-normalized potency of obtained rAAV8 particles, yet the produced viral genome titers were lower compared to state-of-the-art triple transfection. This yield/quality trade-off resulted in similar functional productivity in terms of TU/mL for chromosomal provision of rAAV transgene in the stable D7 cell line and plasmid-based production in HEK293 cells. This observed effect should be further investigated in additional studies with different genome integration loci, with various AAV serotypes and alternative transgenes. To increase the yield obtained by double transfection, a design-of-experiments (DoE) approach could be used to optimize plasmid ratios [15,53]. Increased titers would enable rAAV next generation sequencing (NGS), an orthogonal technique that provides information of packaged DNA in the rAAV particles [18,19].

As next steps towards competitive rAAV production, the rAAV transgene should be integrated with a high copy number, which could enable higher viral genome titers [41]. The remaining genes such as *rep* and *cap* and helper factors should be integrated into the genome as well [42]. This would allow for further increased rAAV titers by process intensification such as perfusion processes [54,55]. Overall, the results in this study provide valuable insights that can be used for the design of genomically integrated rAAV producer lines.

## 4. Materials and Methods

### 4.1. Plasmids

rAAV production plasmids, GOI (pAAV.CMV.PI.EGFP.WPRE.bGH), Rep2Cap8 (pAAV2/8), and helper (pAdDeltaF6) were a gift from James M. Wilson (Addgene plasmid #105530, #112864, and #112867).

The RMCE landing pad sequence was adapted from plasmids kindly provided by Gyun Min Lee (Department of Biological Sciences, KAIST, Daejeon, Republic of Korea) [56]. pSpCas9(BB)-2A-GFP (PX458) was a gift from Feng Zhang (Addgene plasmid #48138) [57], and the primers used to insert guide RNA for AAVS1 into the plasmid can be found in Table S1. The exchange cassette was created by combining parts of four plasmids by Gibson assembly. All primers used in this work were synthesized by Integrated DNA Technologies (Coralville, IA, USA) and are summarized in Table S1. Backbone DNA was amplified from NFκB loxP eGFP plasmid [56], loxP and splice acceptor were used from the landing pad, genomic insulators were taken from the Cre plasmid [56], and neomycin resistance was amplified from pSBtet-BH. pSBtet-BH was a gift from Eric Kowarz (Addgene plasmid #60499) [58]. Amplifications were performed with Q5 polymerase (New England Biolabs (NEB), Ipswich, MA, USA). The GOI fragment was generated by PacI (NEB) restriction of pAAV.CMV.PI.EGFP.WPRE.bGH. The resulting fragments were assembled using NEBuilder^®^ Hifi DNA Assembly Mastermix (NEB) and amplified in NEBStable competent cells (NEB) using heat shock transformation. Plasmid sequence was confirmed using Oxford Nanopore Technologies sequencing (Microsynth, Balgach, Switzerland).

Plasmids used for transfection were prepared using the NucleoBond Xtra Midi Kit (Macherey-Nagel, Düren, Germany).

### 4.2. Cell Culture and Stable Cell Line Generation

Adherent HEK293 cells, ATCC-CRL-1573 (ATCC, Manassas, VA, USA), were maintained in DMEM:F12 (Sigma-Aldrich, St. Louis, MO, USA) with 2 mM L-Glutamine (Gibco, Life Technology Corporation, Grand Island, NY, USA) and 5% FBS (Gibco). Stable cell lines carrying the landing pad were maintained in DMEM:F12 (Sigma-Aldrich) with 2 mM L-Glutamine (Gibco), 10% FBS (Gibco) and 0.5 µg/mL puromycin (Gibco). Stable cell lines carrying the rAAV transgene were maintained in DMEM:F12 (Sigma-Aldrich) with 2 mM L-Glutamine (Gibco), 10% FBS (Gibco), 0.5 µg/mL puromycin (Gibco), and 30 µg/mL G418 (Gibco). Cells were incubated at 37 °C and 8% CO_2_ and maintained in T75 and T175 Nunc EasYFlask (Thermo Fisher Scientific, Waltham, MA, USA).

Integration of the landing pad was achieved by co-transfecting 5 × 10^5^ HEK293 cells with 2.5 µg DNA (landing pad plasmid together with PX458 plasmid targeting AAVS1 locus at a ratio 1:1) with 7.5 µg PEI 25 kDa (Polysciences, Warrington, PA, USA) in a 6-well plate (Corning, New York, NY, USA). After three weeks of antibiotic selection pressure, single cells were sorted with the MoFlo Astrios EQ (Beckman Coulter, Brea, CA, USA) into 384 well plates (Corning) using DMEM:F12 (Sigma-Aldrich) with 2 mM L-Glutamine (Gibco), 10% FBS (Gibco), and 0.5 µg/mL puromycin (Gibco) supplemented with 1X Instigrow HEK (Solentim, Wimborne, UK) and 1X Antibiotic-Antimycotic (Gibco). Cells were gated for mCherry fluorescence. Single cells were upscaled and used for subsequent recombinase-mediated cassette exchange.

RMCE was performed by co-transfection of 2.5 µg DNA (exchange cassette plasmid together with Cre plasmid at a ratio 9:1) with 7.5 µg PEI 25 kDa (Polysciences, Warrington, PA, USA) onto 5 × 10^5^ landing pad carrying cells in a 6-well plate (Corning). Cells were subjected to three weeks of antibiotic selection pressure. Single cell sorting was conducted using the MoFlo Astrios EQ (Beckman Coulter, Brea, CA, USA) into 384 well plate (Corning) using DMEM:F12 (Sigma-Aldrich) with 2 mM L-Glutamine (Gibco), 10% FBS (Gibco), 0.5 µg/mL puromycin (Gibco), and 30 µg/mL G418 (Gibco) supplemented with 1X Instigrow HEK (Solentim, Wimborne, UK) and 1X Antibiotic-Antimycotic (Gibco). Cells were gated for eGFP positive and mCherry negative, as shown in Figure S1A. Growing cells were subsequently passaged into larger vessels until T25 flasks (Thermo Fisher Scientific), where cells were cryopreserved and gDNA samples were taken.

### 4.3. Stable Cell Line Characterization

gDNA was extracted from cell lines using the Monarch Spin Genomic DNA Purification Kit (NEB) and flanking PCR was performed. Therefore, gDNA was amplified with the primers stated in Table S1 for the flanking PCRs.

Copy number determination was performed with ddPCR. gDNA was cleaved with HindIII (NEB). Primer pairs for the different targets—GAPDH, FUTI, LP BB, Puro, 5′ Ins, CMV, WPRE, 3′ Ins—can be found in Table S1. ddPCR was performed using the QX200 system (Bio-Rad, Hercules, CA, USA) with the QX200 ddPCR EvaGreen supermix (Bio-Rad) and PCR mix was prepared according to the manufacturer’s instructions. After droplet generation, PCR was run on a C1000 Touch machine (Bio-Rad) according to the manufacturer’s instructions: 5 min at 95 °C, 40 cycles of 95 °C 30 s, and 60 °C 1 min, followed by 4 °C 5 min, 90 °C 5 min, and 4 °C infinite hold. Each sample was analyzed as three technical replicates. Obtained copies/µL were averaged for the technical replicates and normalized to the housekeeping gene GAPDH for each sample and expressed as percent.

### 4.4. rAAV Production

rAAV productions were performed in 24 well format (Corning). 2.4 × 10^5^ cells were seeded in each well in 500 µL OptiMEM (Gibco). The following day, transfection of rAAV plasmids was performed at a ratio of 2:2:1 GOI, Rep2Cap8, and helper for triple transfection and 2:1 Rep2Cap8 and helper for double transfection. Then, 0.11 µg/cm^2^ DNA and PEIpro (Polyplus, Illkirch-Graffenstaden, France) were transfected in a total volume of 6.7 µL/cm^2^ PBS (Gibco). For rAAV purification, rAAV production in T175 flasks was performed with 22.1 million cells being seeded in 35 mL total OptiMEM medium.

For AdV-assisted rAAV production, 2.4 × 10^5^ cells were seeded into each 24 well. The following day, AdV was diluted in OptiMEM and added to the respective wells at an MOI of 10. Subsequently, well plates were centrifuged at 300 *g* for 5 min to enhance viral entry. Cells with AdV were incubated for two hours before transfection of rAAV plasmids. GOI and Rep2Cap8 plasmids were transfected at a 1:1 ratio.

For wells transfected with siRNA, 3.6 × 10^5^ cells were seeded. Afterwards, the wells treated with siRNA were transfected with 15 pmol Silencer™ eGFP siRNA (Invitrogen, Carlsbad, CA, USA) and 1.5 µL of lipofectamine RNAiMAX (Invitrogen) in a total volume of 50 µL OptiMEM according to the manufacturer’s instructions. The following day, AdV addition and/or plasmid transfections with PEIPro were performed as described above.

72 h post-transfection with PEIPro, rAAV preparations were harvested. Therefore, wells were treated with a 6X concentrated buffer (0.03 M MgCl^2^, 0.3 Tris, 3 (*v*/*v*) Tween-20, and 2.4 M NaCl pH 8) and 50 U/mL SAN HQ (ArcticZymes Technologies, Tromsø, Norway). Lysates were centrifuged at 2000 *g* for 20 min and supernatants were used for rAAV quantification.

Wells designated for flow cytometry analysis were harvested by transferring the media to an Eppendorf tube (Eppendorf, Hamburg, Germany) and washing the wells with 100 µL PBS (Gibco). After incubating the cells with 100 µL trypsin-EDTA (0.25%) (Gibco), the reaction was stopped with 300 µL DMEM + 5% FBS and transferred to the same tube. Cells were centrifuged at 300 *g* for 5 min, the supernatant was discarded, and after resuspension in 500 µL of 4% paraformaldehyde (Sigma-Aldrich), the cells were incubated for 30 min. After centrifugation, cells were washed twice with 500 µL PBS (Corning) and cell pellet was finally resuspended in 180 µL PBS (Corning) and stored at 8 °C until measurement with CytoFLEX S (Beckman-Coulter, Brea, CA, USA). Flow cytometry data was evaluated with Kaluza 2.1 software (Beckman-Coulter).

### 4.5. rAAV Titer Determination

Capsids were quantified using AAV8 Titration ELISA (Progen, Heidelberg, Germany) according to the manufacturer’s instructions.

DNA was prepared from clarified lysates in duplicates by incubating 5 µL of sample with 0.5 µL DNaseI (10 U/µL), 5 µL of the respective 10X concentrated reaction buffer (Sigma-Aldrich), and 39.5 µL nuclease-free water (NFW) (Carl Roth, Karlsruhe, Germany) at 37 °C for 30 min. Subsequently, 3 µL 20 mg/mL Proteinase K (Thermo Fisher Scientific), 1.5 µL 20% SDS solution (Invitrogen), and 5.5 µL NFW were added and again incubated at 37 °C for 30 min, followed by heat inactivation at 95 °C for 20 min. After DNA preparation, samples were diluted 1:50 with low EDTA TE buffer (Thermo Fisher Scientific) and based on the concentration, further dilutions were performed in NFW for ddPCR detection.

Duplex ddPCR was used to quantify ITRs and eGFP simultaneously. The same instruments as described for stable cell line characterization were used. 10 µL ddPCR Supermix for Probes (No dUTP) (Bio-Rad) was mixed with 0.5 µL of respective primer pairs and probes for eGFP and ITR (10 µM) (see Table S1), 0.3 µL MspI (NEB), 5 µL sample, and 1.8 µL NFW. After droplet generation, PCR was performed at 10 min at 95 °C, 40 cycles of 94 °C 30 s, and 55 °C 1 min, followed by 98 °C 10 min and 4 °C infinite hold.

### 4.6. Small-Scale rAAV Purification

Clarified lysate from T175 flask productions was purified by the addition of 50 µL POROS™ CaptureSelect™ AAVX slurry (1:1 with PBS) (Thermo Fisher Scientific). After incubation on a rotator for 2 h at 20 rpm, resin was centrifuged at 300 *g* for 5 min and the supernatant was discarded. Resin was resuspended in 30 mL PBS (Gibco) and incubated for an hour at 20 rpm. After centrifugation at 300 g 5 min, the majority of PBS was removed and around 1 mL of liquid containing the resin was transferred to an AcroPrep 96 well filter plate 1.2 µm (Cytiva, Malborough, MA, USA) and filtrated with vacuum pump PC 3001 VARIO select (Vacuubrand, Wertheim, Germany). Then, 30 µL of 1 M Tris pH 8 (neutralization buffer) was added to the collection deep well plate (Biozym, Hessisch Oldendorf, Germany) and the resin was incubated with 100 µL of 0.1 M sodium acetate pH 3 (elution buffer) for 15 min. Afterwards, elution buffer was filtrated into the collection plate containing the neutralization buffer.

### 4.7. Mass Photometer

For mass photometry, SamuxMP (Refeyn, Waltham, MA, USA) was calibrated with MassFerence™ P2 Calibrant (Refeyn). rAAV samples were diluted in PBS (Gibco) and measured with the MP sample preparation pack (Refeyn) to reach binding counts between 1000 and 4000. Samples were evaluated using automated Gaussian fitting with the Refeyn Discover software 2025 R1.

### 4.8. Backbone ddPCR

DNA isolations from the purified rAAV samples were used for this ddPCR method. For backbone ddPCR, the same settings as described in stable cell line generation were used. The primer targeted AmpR, f1 ori, ori, plasmid upstream, plasmid downstream, gDNA upstream, gDNA downstream, and eGFP (same primer as for rAAV detection) and are listed in Table S1. ddPCR was performed with three biological and three technical replicates. For each biological sample, technical replicates were averaged and obtained copies/µL were normalized to eGFP for each sample.

### 4.9. Potency

5 × 10^4^ HEK293 cells were seeded into a 96 well plate (Corning) and incubated for 2 h. Then, 2 µg/mL mitomycin C (Sigma-Aldrich) was added to each well. After 30 min incubation, purified rAAVs were added in the concentrations 5 × 10^4^, 2.5 × 10^4^, 1 × 10^4^, 4 × 10^3^, and 2 × 10^3^ viral genomes/cell in triplicates. If necessary, rAAVs were diluted in culture media. The final volume per well was 250 µL. The 96 well plate was centrifuged at 300 *g* 5 min and cells were incubated with rAAVs for 48 h. Cells were harvested by discarding the media, washing with 100 µL PBS (Gibco), incubating with 30 µL trypsin (Gibco), and adding 120 µL culture media. Cell suspension was measured using the CytoFLEX S (Beckman-Coulter). Flow cytometry data was evaluated with Kaluza 2.1 software (Beckman-Coulter). HEK293 cells without virus addition served as a benchmark for eGFP fluorescence. Gating strategy of flow cytometry data is depicted in Figure S4. Wells exhibiting between 5 and 25% fluorescent cells were used for potency evaluation to ensure assay linearity. VG/cell dosages outside this requirement were not evaluated. Transducing units/mL were calculated according to the formular used by Emmerling et al. [59].

$$\frac{TU}{mL}=\frac{\%\;gated\times \frac{\begin{array}{c} number\;of\\ cells \end{array}}{well}\times dilution\;factor\;of\;rAAV\;sample}{volume\;of\;rAAV\;added\;[mL]\times 100}$$

As samples varied in viral genome titers, TU/mL was divided by VG/mL and expressed as TU/ million VG to enable comparability of the different samples. The same calculation method was applied to each tested sample.

### 4.10. Statistical Analysis

Data was analyzed with GraphPad Prism 10 (GraphPad, Boston, MA, USA). Student’s *t*-test, one-way ANOVA with Tukey’s multiple comparison test, and outlier detection using the ROUT method (Q = 10%) were performed. Level of significance was set to *p* ≤ 0.05.

## Data Availability

The original contributions presented in this study are included in the article/Supplementary Materials. Further inquiries can be directed to the corresponding author.

## Supplementary Materials

The following supporting information can be downloaded at https://www.mdpi.com/article/10.3390/ijms27125538/s1.
