# Supplementary material for: Comparative Analysis of rAAV Production from Plasmid-Encoded Versus Chromosomally Integrated rAAV Transgene in HEK293 Cells
Source: Int J Mol Sci. 2026 Jun 18;27(12):5538. doi: 10.3390/ijms27125538 (PMC13299565; doi:10.3390/ijms27125538)
Supplement: Supplementary file 1 [file ijms-27-05538-s001.zip › ijms-4304890-supplementary.pdf]

Table S1. Primer pairs used for cloning, flanking PCRs, copy number determination of integrated cassette, rAAV viral genome quantification and plasmid backbone contamination detection.

Target	Forward primer (5′-3′)	Reverse primer (5′-3′)
AAVS1 gRNA	GTGGGGTACCCTAAGAACTTG	CCACTAAGGCAATTGGGGT
plasmid backbone from NFkB loxP eGFP plasmid	TACCGTCGACCTCTAGCTAGAGCTTATAACTTCGTATAGGATACTTTATAC	CAGGTATAAATGGGCTCG
loxP and puromycin resistance from RMCE landing pad	ATTATCGCGAGCCCATTTATACCTGGCAAGCCCGGTGCCTGAA	ACGATTCGGGATCAAGCCATAGAGCCCACCG
genomic insulators from Cre plasmid	GCTCTATGGCTTGATCCCGAATCGTTTAAAC	CGCGCAGCCTAATTAAGGCCTTAATGGTACCAATTGAGAGGTAGCTGAAG
genomic insulators from Cre plasmid	GAGCGAGCGAGCGCGCAGCCTTAATTCTAGAGGTCGTCAGCTATCCTGC	CACCCTAACTGACACACATTCCACATGCAATAGCAAGGACCAG
neomycin resistance from pSBtet-BH	TGTGGAATGTGTGTCAGTTAG	AAGCTCTAGCTAGAGGTC
transgene upstream flanking PCR	CAGTCCTCCTTACCATCC	TCGTCCTTGAAGAAGATGG
transgene downstream flanking PCR	TTTCTGGATTCATCGACTGTGG	CCACTAAGGCAATTGGGGT
GAPDH	CATCCTGGGCTACACTGAG	CAGCGTCAAAGGTGGAGG
FUTI	AGCAACGGCATGGAGTGGTGTA	AAGCCGAAGGTGCCAATGGTCA
LP BB	CACAACGAGGACTACACCATCG	TCGAGGCTGATCAGCGGTTTAA
Puro	AAGCCCGGTGCCTGAATAAC	CTAGAAGGCACAGTCGAGGC
5′Ins	CTCGTCAGCTATCGTCAG	GGTAGCTGAAGCTGCTG
CMV	AGGAAGATCGGAATTCGCCC	ACCGTAAGTTATGTAACGCGGA
WPRE	CAATTCCGTGGTGTTGTCGG	CCGAAGGGACGTAGCAGAAG
3′Ins	ATGGTCGTCAGCTATCCTGC	CCCCTAGTGGTGTCCAGAA
eGFP	GAACCGCATCGAGCTGAA	TGCTTGTCGGCCATGATATAG
eGFP probe	6-FAM-TTGCCGTCC-ZEN-TCCTTGAAGTCGAT-Iowa Black FQ	
ITR	GGAACCCCTAGTGATGGAGTT	CGGCCTCAGTGAGCGA
ITR probe	HEX-CACTCCCTC-ZEN-TCTGCGCGCTCG-Iowa Black FQ	
ampR	TCCTTGAGAGTTTTCGCCCC	CCGGCGTCAATACGGGATA
f1 ori	TCCTTTCGCTTTCTTCCCTTCC	GCCGTAAAGCACTAAATCGGAAC
ori	TTGGAGCGAACGACCTACAC	CCGCTTACCGGATACCTGTC
plasmid upstream	CCTTAATTAACCTAATTCACTGGCCGT	TTCGCTATTACGCCAGCTG
plasmid downstream	GAGTTAGCTCACTCATTAGG	CCTAATTAAGGCCTTAATTAAATCTG
gDNA upstream	CTCGTCAGCTATCGTCAG	GGTAGCTGAAGCTGCTG
gDNA downstream	TAATGGTCGTCAGCTATCC	GTTTGATGAACTACTTCTGACC
eGFP	GAACCGCATCGAGCTGAA	TGCTTGTCGGCCATGATATAG

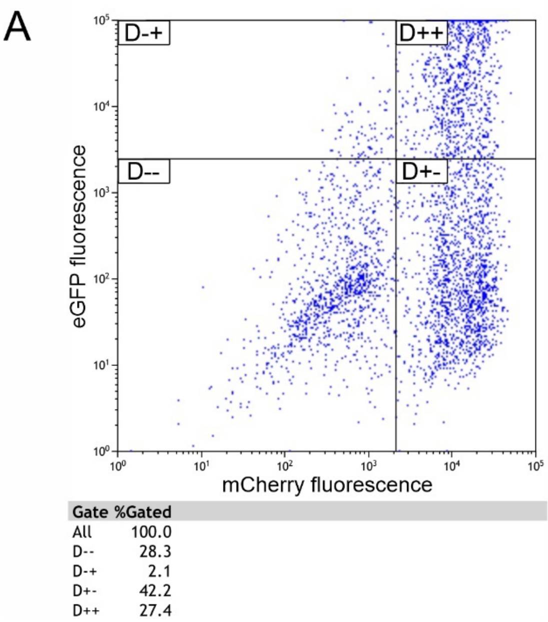

B

normalized to GAPDH

	K5	D7	K9
**GAPDH**	100%	100%	100%
**FUTI**	73%	108%	84%
**LP BB**	NA	NA	NA
**Puro**	28%	32%	31%
**5' INS**	26%	32%	33%
**CMV**	24%	31%	33%
**WPRE**	27%	34%	34%
**3' INS**	24%	31%	32%

Figure S1. Characterization of stable cell lines carrying an rAAV transgene. (**A**) Flow cytometry assisted single cell sorting. Cells were sorted for mCherry negative (x axis) and eGFP positive (y axis), marked by gate D-+. (**B**) Digital droplet PCR (ddPCR) on genomic DNA to assess genomic stability after experiments were finished (40 passages). Measurements in technical triplicates (n = 3) and mean is depicted. Two housekeeping genes were included; values were normalized to GAPDH and expressed as percent. Primer sets targeted: the landing pad backbone (LP BB; yellow), puromycin resistance (Puro; orange) that amplifies in both the landing pad and the integrated cassette, and four regions specific to the integration cassette (blue).

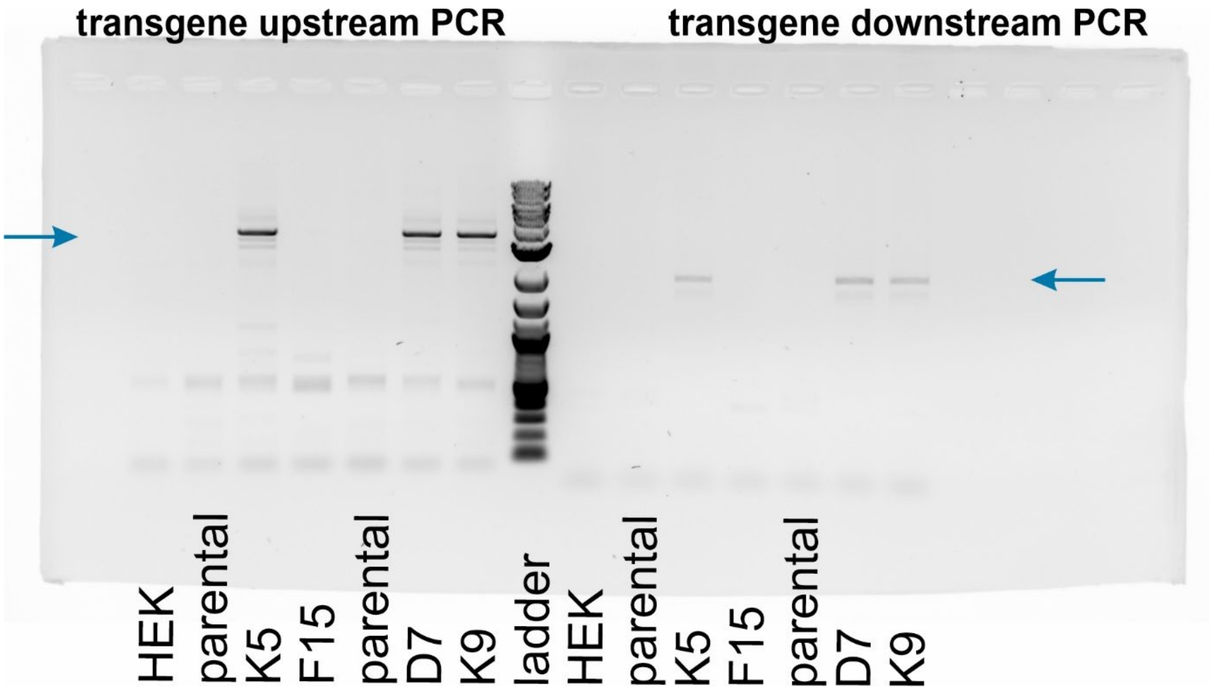

Figure S2. Uncropped flanking PCR gel depicted in Figure 1B to verify site-specific integration in genetically unmodified HEK293 cells, parental HEK293 cells harboring the loxP landing pad, and four derived stable clones carrying the exchange cassette. One primer annealed in the unmodified genomic region and the other within the introduced cassette. Expected amplicons were 3,650 bp (transgene upstream) and 2,000 bp (transgene downstream) and are indicated by arrows. 1 kb plus DNA ladder (NEB) was used.

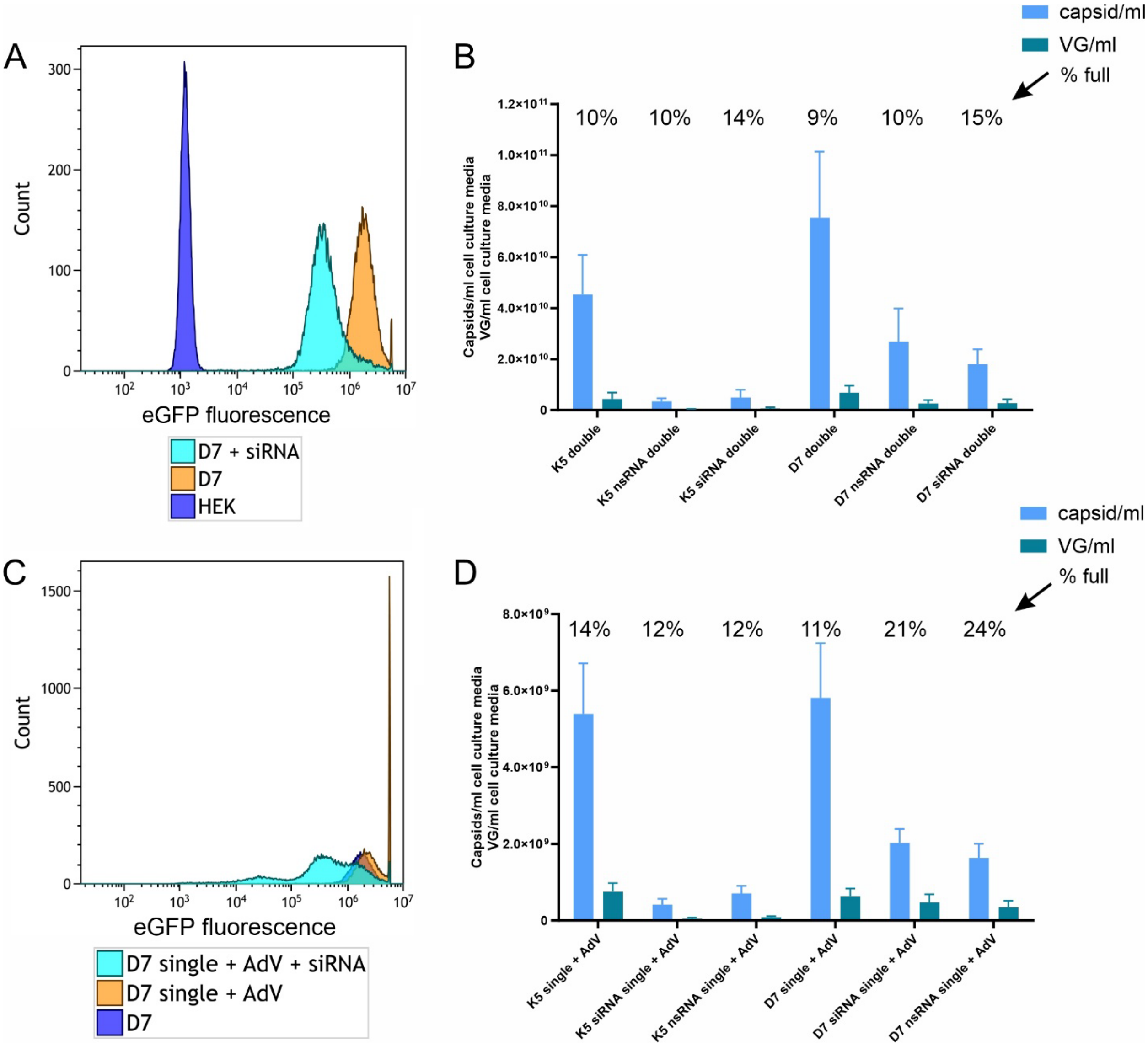

Figure S3. rAAV production with siRNA targeting eGFP mRNA. (**A**) eGFP fluorescence of original HEK293 cells and stable D7 clone with and without siRNA. (**B**) Plasmid based rAAV production with siRNA. rAAV production in stable cell lines K5 and D7 by co-transfection of Rep2Cap8 and helper plasmid, as well as with prior transfection of siRNA or control nonsense RNA (nsRNA). Capsids were measured with ELISA and viral genome (VG) titers were obtained by ddPCR ddPCR and expressed per ml cell culture media. % full represents the percentage of rAAV particles filled with a viral genome calculated with the means of capsid and VG titers. The column and error bars represent the mean and SD of n = 5 biological replicates. Outliers were detected with ROUT method (Q=10%). (**C**) eGFP fluorescence of stable D7 clone with and without Adenovirus infection and siRNA. (**D**) rAAV production with helper AdV virus and siRNA. rAAV production in stable cell lines K5 and D7 by transfection Rep2Cap8 and AdV (single transfection + AdV), as well as with prior transfection of siRNA or control nonsense RNA (nsRNA). Capsids were measured with ELISA and viral genome titers were obtained by ddPCR and expressed per ml cell culture media. % full represents the percentage of rAAV particles filled with a viral genome calculated with the means of

capsid and VG titers. The column and error bars represent the mean and SD of n = 5 biological replicates. Outliers were detected by ROUT method (Q=10%).

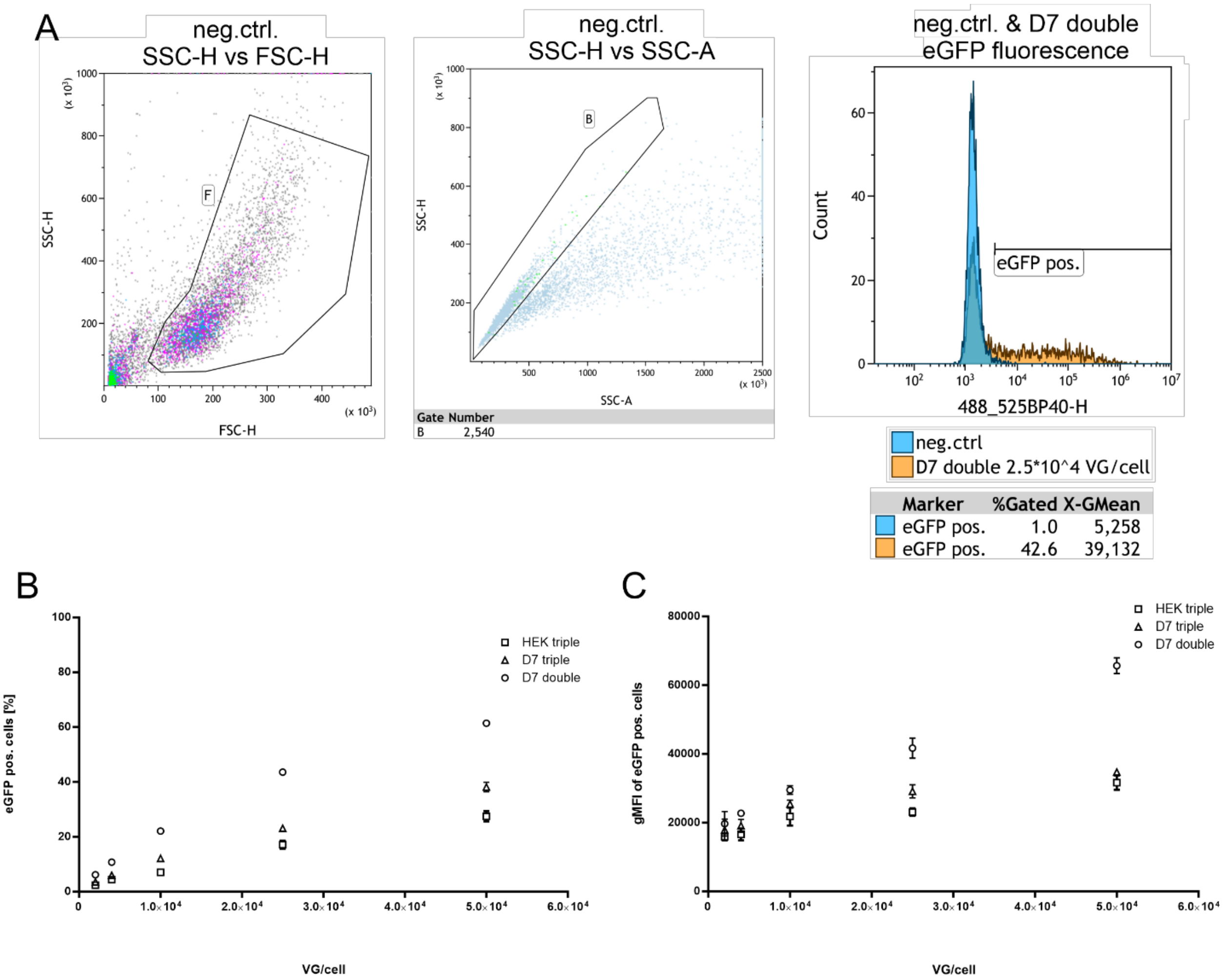

Figure S4. Potency assay with purified rAAVs from HEK293 and D7 triple transfection and D7 double transfection. Different concentrations of rAAVs carrying a CMV-eGFP-WPRE sequence were incubated with HEK293 cells for 48 hours. (**A**) Gating strategy for detecting eGFP fluorescence in potency assay using flow cytometry. Selection of cells using side and forward scatter (SSC and FSC). Selection of single cells based on side scatter height (SSC-H) and area (SSC-A). eGFP fluorescence histogram (488 nm laser) of negative control and fluorescent sample (rAAVs from D7 double transfection at 2.5 x 10^4 VG/cell). A gate detecting percent eGFP positive cells was set to 1% of cells in the negative control. Fluorescence intensity was measured as geometric mean of fluorescence in the gate (X-GMean). (**B**) eGFP positive cells were measured by flow cytometry and depicted for each tested dosage of VG per cell, namely 2 x 10^3, 4 x 10^3, 1 x 10^4, 2.5 x 10^4 and 5 x 10^4. (**C**) Similarly, geometric mean fluorescent intensity (gMFI) of eGFP positive cells were depicted. The error bars represent the mean and SD of n = 3 replicates.
